# Organizing Pneumonia as a Pulmonary Sequela of Post-COVID-19 Syndrome in a Patient in Trinidad: A Case Report

**DOI:** 10.7759/cureus.50148

**Published:** 2023-12-07

**Authors:** Nishtha Mohan, Dominic Dalip, Shiva Jaggernauth

**Affiliations:** 1 Internal Medicine, The University of the West Indies, St. Augustine, TTO; 2 Internal Medicine, Leicester Royal Infirmary, Leicester, GBR; 3 Respiratory Medicine, Apley Medical Clinic, San Fernando, TTO; 4 Pulmonary Medicine, Southern Medical Clinic, San Fernando, TTO

**Keywords:** idiopathic interstitial pneumonia, interstial lung disease, covid 19, long covid-19 pulmonary sequelae, cryptogenic organizing pneumonia, post-covid-19 organizing pneumonia

## Abstract

Cryptogenic organizing pneumonia (COP) is a form of idiopathic interstitial pneumonia that commonly presents with exertional dyspnea. The mainstay diagnostic criterion is with histopathological confirmation alongside excluding secondary causes of interstitial lung disease. The COVID-19 pandemic left many mysteries regarding the long-term sequelae of this disease. We explore a case of post-COVID-19 syndrome organizing pneumonia (PCOP) in a patient presenting with new-onset respiratory symptoms seven weeks after recovery from COVID-19 infection. Upon further review of the literature, there were no published case reports on PCOP in Trinidad and Tobago. We describe a case of PCOP presented at Apley Medical Clinic, Trinidad, and Tobago, West Indies, with the aim of increasing awareness of this condition to allow for early identification and effective management.

## Introduction

Cryptogenic organizing pneumonia (COP) is a form of idiopathic interstitial pneumonia that commonly presents with exertional dyspnea [1]. Our patient’s clinical and radiological findings were compatible with a diagnosis of COP. However, a definitive diagnosis can only be established with histopathological confirmation [1]. As we are teetering on the precipice of the coronavirus pandemic’s end, we anticipate many discoveries regarding COVID-19 sequelae. Herein, we explore a case of post-COVID-19 syndrome organizing pneumonia (PCOP) where our patient presented with new-onset respiratory symptoms seven weeks after recovery from COVID-19 infection and was subsequently diagnosed with COP, following extensive diagnostic evaluation to rule out other secondary causes. In the absence of a formal set of guidelines in the management of COP, the general consensus advises the use of corticosteroid therapy with documented evidence of its favorable therapeutic response [1,2]. However, the literature is even more limited regarding the novel PCOP and consequently, its management is currently at the clinician’s discretion, with many undertaking the same therapeutic steps as in COP [2-5]. This highlights the unprecedented need for further research on PCOP to adequately address the existing gaps in knowledge such as optimal management strategies with the goal of reducing morbidity and mortality of this post-COVID-19 pulmonary sequela [3]. Upon further review of the literature, there were no published case reports on PCOP in Trinidad and Tobago. We describe a case of PCOP presented at Apley Medical Clinic, Trinidad, and Tobago, West Indies, with the aim of increasing awareness of this condition to allow for early identification and effective management.

## Case presentation

A 70-year-old male presented to the chest clinic with a two-week history of low-grade fever, exertional dyspnea, and dysuria. He had a medical history of hypertension, ischemic heart disease, prostate cancer treated with brachytherapy, and most recently, confirmed COVID-19 infection seven weeks ago that did not require hospitalization. The patient reported that his symptoms were progressively worsening despite treatment with a five-day course of amoxicillin/clavulanate as prescribed by his general practitioner who maintained a high suspicion of urinary tract infection. Ciprofloxacin 500 mg orally twice daily for one week was used after his amoxicillin/clavulanate course and the dysuria was subsequently resolved. However, the patient complained of persistent exertional dyspnea accompanied by an occasional non-productive cough. He denied hemoptysis.

He had a modified Medical Research Council (mMRC) score of 2 and his peripheral oxygen saturation (SpO_2_) was 92% on room air. Repeat nasopharyngeal SARS-CoV-2 reverse transcriptase polymerase chain reaction (RT-PCR) was negative. Physical examination was remarkable for decreased air entry and bilateral crackles in all zones on auscultation. Laboratory investigations showed an elevated inflammatory response (Table 1). Chest radiograph (CXR) revealed the new emergence of bilateral peri-bronchial consolidation as compared to previous films. The patient was prescribed portable oxygen. Pulmonary function test (PFT) showed moderate restrictive ventilatory defect (Table 2).

**Table 1:** Results of laboratory investigations in the present study WBC, white blood cells; ESO, eosinophils; HB, hemoglobin; PLT, platelets; ESR, erythrocyte sedimentation rate; CRP, c-reactive protein; BUN, blood urea nitrogen; CR, creatinine; NA, sodium; K, potassium; Cl, chloride.

Investigation	Units	Normal Range	Pre-Corticosteroid Therapy	Post-Corticosteroid Therapy
WBC	10^3^/uL	4.5-11.0	10.2	15.2
EOS	10^3^/uL	0.0-0.5	0.0	0.0
HB	g/dL	13.5-17.5	14.5	14.9
PLT	10^3^/uL	150-400	440	275
ESR	mm/h	0.0-22.0	105	62
CRP	mg/dL	0.0-10.0	118	3.3
BUN	mg/dL	6.0-24.0	14.0	15.1
CR	mmol/L	0.7-1.3	0.8	0.7
NA	mmol/L	135-145	138	139
K	mmol/L	3.5-5.1	3.7	4.7
Cl	mmol/L	98-112	102	99

**Table 2:** Pulmonary function test results of the present study Pred, predicted; LLN, lower limit of normal; Chg, change; Abs, absolute; FVC, forced vital capacity; FEV, forced expiratory volume; FEF, forced expiratory flow; PEFR, peak expiratory flow rate; FIF, forced inspiratory flow; MVV, maximum voluntary ventilation; TLC, total lung capacity; VC, vital capacity; RV, residual volume; FRC, functional residual capacity; SVC, slow vital capacity; IC, inspiratory capacity; ERV, expiratory reserve volume; DLCO, diffusion capacity of lung for carbon monoxide; VA, alveolar ventilation; MIP, maximal inspiratory pressure; MEP, maximal expiratory pressure; CPF, cough peak flow.

Spirometry	Units	Pred	LLN	Best	%Pred
FVC	L	3.77	2.65	2.48	66
FEV1	L	2.96	2.12	2.07	70
FEV1/FVC	%	79	71	73	105
FEV6	L	—		2.44	
FEF25-75[iso]	L/s	2.88	1.21	2.85	99
PEFR	L/s	7.10	3.21	7.94	112
FEF50	L/s	3.22	1.09	4.33	134
FIF50	L/s			4.07	
FEF50/FIF50				1.06	
MVV	L/m	112.8	56.0	113.7	101
Lung Volumes					
TLC	L	5.92	4.46	3.28	55
VC	L	3.77	2.65	2.54	67
RV	L	2.15	1.39	0.74	34
RV/TLC	%	35	26	23	66
FRC	L	3.12	1.66	1.38	44
SVC	L	3.77	2.65		
IC	L	2.80	1.34	1.90	68
ERV	L	0.97		0.64	66
Diffusing Capacity					
DLCO	mL/min/mmHg	20.07	11.87	8.86	43
DLCO [Hb]	mL/min/mmHg	20.07	11.87	9.17	46
DLCO/VA	mL/min/mmHg	3.63	1.79	2.81	77
VA [BTPS]	L	6.01	4.40	3.26	54
Muscle Force					
MIP	cmH_2_O	70	19	75	107
MEP	cmH_2_O	117	61	78	67
Cough Peak Flow					
CPF	L/s	12.13	8.63	6.81	56
CPF 60	L/min	728	518	409	56

**Figure 1:**
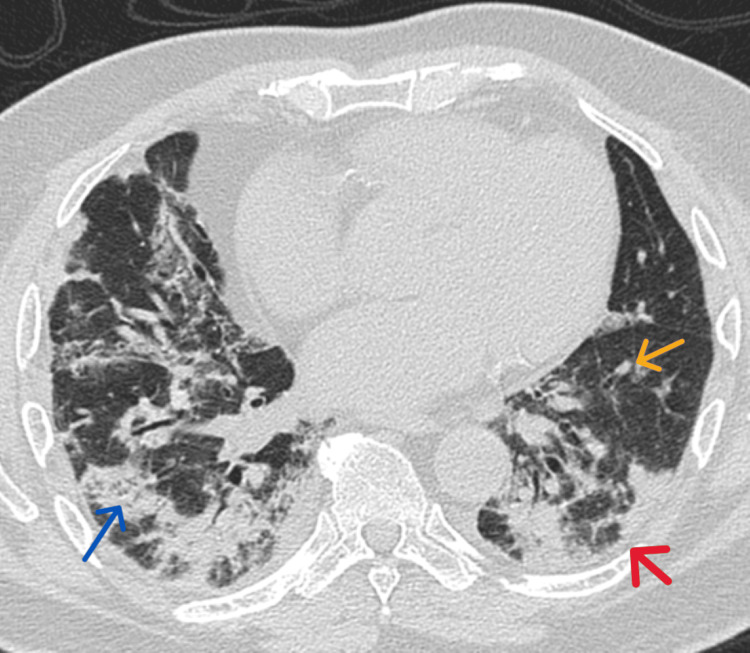
CT chest showing findings consistent with cryptogenic organizing pneumonia Blue arrow: consolidation, yellow arrow: nodules, pink arrow: perilobular pattern.

During the process of diagnostic evaluation, the patient returned to the clinic complaining of dyspnea and having high titers of inflammatory markers. His SpO_2_ deteriorated to 92% on 2 liters of oxygen. Contrast computed tomography (CT) of the chest revealed a pattern compatible with organizing pneumonia (OP) in the absence of fibrosis (Figure 1). These findings include consolidations, nodules, and a perilobular pattern. Further laboratory investigations showed neither eosinophilia nor antineutrophil cytoplasmic antibody (ANCA immunoblot). Additionally, there was a negative bacterial and viral pathogen work-up. The patient declined confirmatory lung biopsy due to health concerns and a clinic-radiological diagnosis of COP secondary to post-COVID-19 infection was made by the pulmonologist. Prednisolone 30 mg orally daily was immediately commenced and within one week, the patient demonstrated a considerable clinical response to the corticosteroid therapy. His mMRC score was 0, his SpO_2_ was 95% on room air and his chest was clear on physical examination. The dose of prednisolone was subsequently reduced to 15 mg orally daily for one month.

After this, he reported restoration of his normal exercise tolerance. Additionally, his SpO_2_ had improved to 97% on room air, and a repeat chest radiograph showed a reduction in the size of the shadow in the right lung with the disappearance of that in the left. Laboratory investigations revealed decreased inflammation (Table 1). These findings were suggestive of resolving COP secondary to post-COVID-19 infection and his prednisolone dose was further tapered to 10 mg daily for one month. There have been no reported relapses in this patient to this date.

## Discussion

OP is a form of interstitial pneumonia formerly known as bronchiolitis obliterans organizing pneumonia [1,2]. The pathological criteria stipulate organized polypoid granulation inflammatory tissue in the distal bronchiole airways, respiratory bronchioles, alveolar ducts, and alveoli in addition to negative findings of extensive interstitial fibrosis, traction bronchiectasis, and histologic honeycombing as historically described by Epler and Colby [6].

Variants of OP have since emerged and subsequently been studied. Cryptogenic OP is reserved for its primary or idiopathic form where no identifiable cause has been isolated [1]. Secondary OP refers to the presence of an attributable cause or defined association of which documented etiologies include infections - viral (e.g. herpesvirus), bacterial (e.g. *Chlamydia pneumoniae*), fungal (e.g. *Cryptococcus neoformans*), parasitical (e.g. *Plasmodium vivax*) - drug toxicity (e.g. amiodarone), radiation (e.g. breast cancer therapy), inhalation injury (e.g. aspiration), hematological cancers, transplantation, connective tissue disease, and inflammatory bowel disease among others [1,7,8]. Moreover, rapidly progressive OP is a rare but fatal variant that responds poorly to standard therapy with a presentation similar to adult respiratory distress syndrome with progression to respiratory failure necessitating tracheal intubation and mechanical ventilation [7].

The symptomatology of COP has been described, according to one study, as non-productive cough (71%), exertional dyspnea (62%), and fever (44%), features also present in our patient [1]. Another study suggested that fever may be indicative of secondary OP [4]. Additionally, our patient showed no clinical response to antibiotic therapy which heightened the clinical suspicion of OP [1].

The most common abnormality on chest auscultation in patients with OP was crackles, as was present in our patient [1]. Although non-specific, increased levels of ESR, CRP, and leukocytes can be supplementary findings in the blood investigations of patients with OP, also found in our patient, indicating an elevated inflammatory response [1,4]. PFT often shows restrictive dysfunction with reduced diffusing capacity for carbon monoxide in keeping with the present study [1,3]. Our patient’s CXR demonstrated bilateral opacities which are evident in OP [1]. The reverse halo or atoll sign is a relatively specific yet uncommon finding on CT suggestive of COP, observed in fewer than 5% of cases [1,5]. Other radiological findings include ground-glass opacities, consolidations, crazy-paving pattern, and reticulation (Table 3) [2,4]. Histopathological confirmation of intraluminal plugs of loose connective tissue, inflammatory infiltrate in the intervening alveolar walls with the lung architecture intact in the absence of established fibrosis, is required for the definitive diagnosis of OP [1]. However, performing a lung biopsy is frequently impractical in the clinical setting [1]. Therefore, taking our patient’s clinical presentation and radiological findings into account together with his negative viral and bacterial pathogen work-up, a clinic-radiological diagnosis of COP was made.

Our patient’s diagnosis of COP that developed seven weeks after complete clinical recovery of COVID-19 infection in concert with our knowledge of SARS-CoV-2 as one of the many documented etiological agents of secondary OP lends plausibility to COP as an under-recognized pulmonary sequela of PCOP [1,9]. The National Institute for Health and Care Excellence (NICE) has defined “Post-COVID-19 syndrome” as sequelae that develop during or after a SARS-CoV-2 infection and persist for more than 12 weeks, encompassed in the term “long COVID-19” [3]. While the literature currently acknowledges that abnormalities of the lung’s interstitium and microvasculature are pulmonary sequelae of post-COVID-19 syndrome, much less is known about the intricacies of these said interstitial lung abnormalities, such as OP [10]. Both fibrotic-like and non-fibrotic radiographic patterns have been identified in post-COVID-19 syndrome patients [3]. A cohort study of patients with post-COVID-19 syndrome found that features of OP dominated the findings in those with non-fibrotic interstitial lung disease [3,10].

There is an unprecedented need to identify PCOP in order to ensure early diagnosis and effective management of patients with post-COVID-19 sequelae. A high index of suspicion for PCOP should exist with new-onset respiratory symptoms, particularly dyspnea or dry cough, refractory to antibiotic therapy in patients with a history of COVID-19 infection [5].

The mainstay of COP treatment, although empirical, is corticosteroids with protocols ranging from the long-established high-dose prednisolone at 60 mg orally daily (1 mg/kg/day) for one to three months tapered to 40 mg for three months then to 10 mg daily for one year as initially proposed by Epler to more recent lower-dose regimens of shorter durations [1,2,7,8]. In the absence of standardized therapies for post-COVID-19 interstitial lung abnormalities to date, its use has been extended to the treatment of PCOP, with the rationale being similarities in the pathogenesis of both conditions [2,4,5]. This therapy has proved efficacious in several published case series [2,5,9]. Moreover, a similar outcome was achieved in a single-center prospective observational study whereby post-COVID-19 patients presenting with dyspnea and evidence of OP exhibited clinical and radiological resolution following corticosteroid therapy [5,10]. In contrast to the preponderance of high-dose prednisolone regimens documented in the literature, the present study opted for a lower dose - 30 mg orally daily for one week and after subsequent clinical improvement, the dose was tapered to 15 mg orally daily for one week then 10 mg orally daily for one week [2,4]. Our patient demonstrated a favorable therapeutic response similar to that achieved by other studies that employed lower doses [4,10]. Curiously, one study illustrated similar effectiveness with both high and low doses of prednisone [4]. Notwithstanding, it is of paramount importance that further research be conducted in order to fill the current gap in the knowledge as optimal doses and duration of treatment are unknown and, thus, left at the discretion of the clinician [2,4,5].

In cases of COP refractory to steroid therapy, corticosteroid-sparing agents and/or cytotoxic agents have been used [1,6]. Taking into consideration its variable success, corticosteroid-sparing agents such as macrolides with anti-inflammatory effects, namely erythromycin or clarithromycin, may be used for a minimum of three to six months [1]. Another alternative is cytotoxic or immunosuppressive therapy such as azathioprine and cyclophosphamide [1]. However, cyclophosphamide is usually reserved for cases of rapidly progressive OP and given in addition to high-dose prednisolone [7].

It is noteworthy of mentioning that COP relapses have been reported in 40% of cases, most occurring within one year after the initial episode [4,8]. A study of patients with recurrent COP identified two possible predictors of multiple relapses: delayed treatment of the initial episode and increased markers of cholestasis (elevated gamma-glutamyltransferase and alkaline phosphatase) [7,8]. The treatment involves reinstitution of corticosteroid therapy, administered intravenously if rapidly progressive, in addition to pulmonary rehabilitation and, in rare events, lung transplantation [6,8]. Pulmonary rehabilitation has a role in managing the mid-to-late phase of OP after the commencement of corticosteroid therapy while lung transplantation is an option typically reserved for patients unresponsive to first-line treatment [6]. To date, data are scarce regarding relapses of PCOP.

In the future, anti-fibrotic therapy may contribute to the treatment of PCOP. Although approved for idiopathic pulmonary fibrosis, the role of anti-fibrotic agents, pirfenidone and nintedanib, in the management of PCOP has yet to be fully elucidated [3,4]. They exhibit anti-inflammatory and cytoprotective effects which may help prevent the progression of inflammatory change to irreversible pulmonary fibrosis, a well-known sequela of COVID-19 [3,4,10].

The major limitation of this case report is the lack of histopathological confirmation of the diagnosis of COP due to the patient’s frail health status. Another limitation is the ambiguity surrounding the dose and duration of corticosteroid therapy as there is currently no published set of guidelines with respect to the treatment of PCOP.

## Conclusions

In conclusion, we report a case of PCOP in Trinidad, West Indies, that was not previously reported in the literature. Clinicians should maintain a high index of suspicion for PCOP in patients with new-onset respiratory symptoms, particularly dyspnea or dry cough, refractory to antibiotic therapy with a history of COVID-19 infection. Treatment is not standardized due to limited published data; however, the existing case series and cohort study demonstrate a favorable therapeutic response to corticosteroid therapy. However, there remains a great need for further research on optimal management of PCOP.
